# Cerebral Blood Flow Modulation by Basal Forebrain or Whisker Stimulation Can Occur Independently of Large Cytosolic Ca^2+^ Signaling in Astrocytes

**DOI:** 10.1371/journal.pone.0066525

**Published:** 2013-06-13

**Authors:** Norio Takata, Terumi Nagai, Katsuya Ozawa, Yuki Oe, Katsuhiko Mikoshiba, Hajime Hirase

**Affiliations:** 1 Laboratory for Neuron-Glia Circuit, RIKEN Brain Science Institute, Wako, Saitama, Japan; 2 Laboratory for Developmental Neurobiology, RIKEN Brain Science Institute, Wako, Saitama, Japan; 3 Japan Science and Technology Agency, ICORP and SORST, Calcium Oscillation Project, Saitama, Japan; 4 Saitama University Brain Science Institute, Saitama, Saitama, Japan; University of Nebraska Medical Center, United States of America

## Abstract

We report that a brief electrical stimulation of the nucleus basalis of Meynert (NBM), the primary source of cholinergic projection to the cerebral cortex, induces a biphasic cerebral cortical blood flow (CBF) response in the somatosensory cortex of C57BL/6J mice. This CBF response, measured by laser Doppler flowmetry, was attenuated by the muscarinic type acetylcholine receptor antagonist atropine, suggesting a possible involvement of astrocytes in this type of CBF modulation. However, we find that IP3R2 knockout mice, which lack cytosolic Ca2+ surges in astrocytes, show similar CBF changes. Moreover, whisker stimulation resulted in similar degrees of CBF increase in IP3R2 knockout mice and the background strain C57BL/6J. Our results show that neural activity-driven CBF modulation could occur without large cytosolic increases of Ca2+ in astrocytes.

## Introduction

Prolonged activation of the nucleus basalis of Meynert (NBM), the primary source of cholinergic projection to the cerebral cortex, has been reported to cause significant increases in cerebral blood flow (CBF) in rodents [1]. While the NBM also gives rise to GABAergic and glutamatergic projections to the cerebral cortex [2], [3], the NBM-driven increase of CBF has been described to be dependent on the synergistic effect of muscarinic and nicotinic acetylcholine receptors (mAChRs and nAChRs, respectively) [1], [4]. Lately, several groups reported that astrocytes, a glia cell type that contacts vasculature and ensheath synapses, modulate local CBF via intracellular Ca^2+^ signaling [5]–[8]. Considering that cortical astrocytes express mAChRs [9], [10] and *in vivo* activation of the NBM leads to mAChR-dependent Ca^2+^ surges in astrocytes [11], cholinergic modulation of CBF via astrocytic Ca^2+^ surges is conceivable. Similarly, prolonged sensory stimulation has been demonstrated to increase somatosensory cortical CBF [12] and induce Ca^2+^ surges of astrocytes [13], suggesting a similar mechanism of CBF modulation via astrocytic G-protein coupled receptors.

The source of Ca^2+^ for astrocytic Ca^2+^ surges is predominantly internal stores such as the endoplasmic reticulum. The activation of inositol trisphosphate receptor type 2 (IP_3_R2), an IP_3_ receptor type specifically expressed in astrocytes in the central nervous system [14], is critical for astrocytic Ca^2+^ surges in the hippocampus [15] and cerebral cortex [11]. In the current study, we asked if large cytosolic Ca^2+^ surges in astrocytes is required in neural activity-driven CBF changes using IP_3_R2 knockout mice (IP_3_R2-KO) [16], in which cytosolic Ca^2+^ response is absent in astrocytes upon NBM- or whisker stimulation while local field potential response to these stimuli are apparently normal [11].

## Results

**Figure 1:**
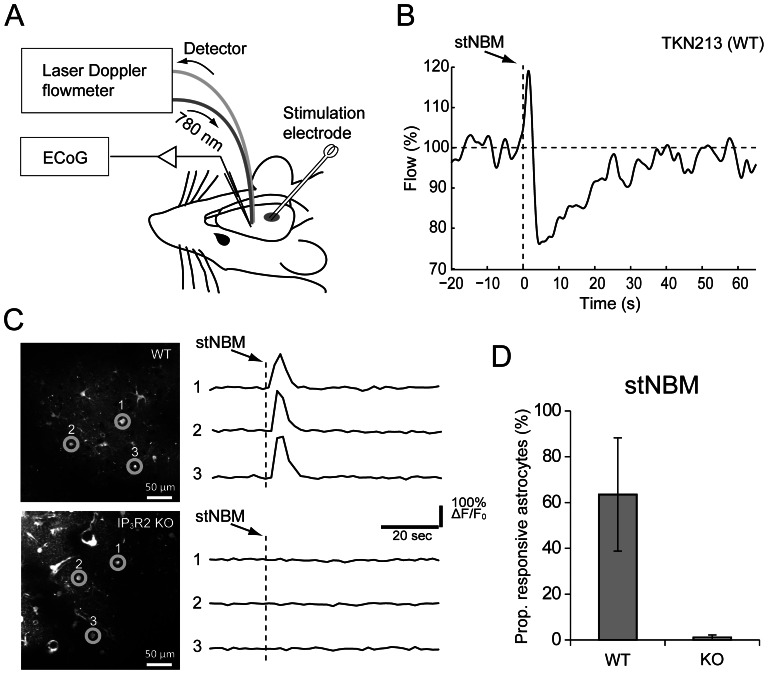
Overview of the stNBM experiment. (**A**) Sketch for experimental set up. A laser Doppler probe is placed above the thinned skull at the primary somatosensory cortex. A bipolar stimulation electrode is inserted to target the nucleus basalis of Meynert (NBM) in the ipsilateral side. (**B**) An example trace of laser Doppler flowmetry from a wild type mouse in response to a single train NBM stimulation (stNBM, arrow). (**C**) *In vivo* two-photon imaging of Fluo-4 AM loaded astrocytes in the somatosensory cortex of C57BL/6J (WT, upper panels) and IP_3_R2-KO (lower panels). (**D**) Proportion of barrel cortex astrocytes that elicited Ca^2+^ elevations upon stNBM.

In the present study, we compared NBM-triggered CBF changes between IP_3_R2-KO mice and their background strain C57BL/6J mice. In C57BL/6J mice, delivery of a brief single train stimulation of the NBM (stNBM, 100 Hz, 0.5 ms pulse width, 50 pulses, 200 µA) resulted in an immediate increase of CBF, followed by a transient decrease that overshot the baseline (Figure 1A&B). Interestingly, large changes of blood flow were observed in the first 10 s, which roughly corresponds to the period of activated (desynchronized) LFP pattern [11]. After this point, the blood flow slowly tapered off to baseline in ∼25 s. In a separate set of experiments, we observed that 64±25% of the imaged astrocytes elicit Ca^2+^ surges induced by stNBM whereas astrocytic Ca^2+^ surges were rare (1±1%) in IP_3_R2-KO (Figure 1 C&D, n = 3 animals for each genotype, p<0.05).

**Figure 2:**
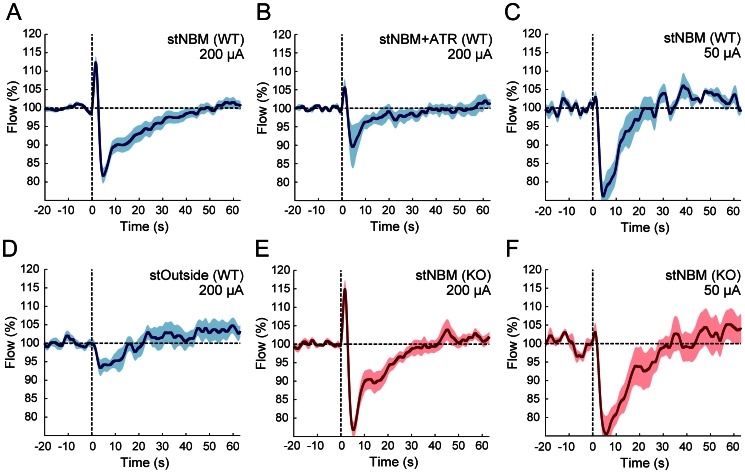
Averaged traces of laser Doppler flowmetry in C57BL/6J and IP_3_R2-KO mice. Results for C57BL/6J and IP_3_R2-KO mice are represented in blue and red traces, respectively. Each experimental condition has N>5 animals. Upon 200 µA stNBM, the cerebral blood flow (CBF) showed an immediate increase, followed by a transient decrease that overshot the baseline both in WT (**A**) and KO (**E**). CBF change by the stNBM is attenuated by the muscarinic receptor antagonist atropine (ATR) (**B**). Weak stNBM stimulation (50 µA) resulted in negative laser Doppler flowmetry signal (**C**) and similar changes were observed in KO (**F**). Stimulation outside the NBM failed to induce CBF increase (**D**). Shaded areas represent s.e.m.

The stNBM-triggered CBF peak was reached at 1.56±0.07 s (n = 17 animals, Figure 2A) and the peak value was 113.0±1.7% (p<0.001, *vs.* prestimulus period). The following negative peak was reached at 6.90±1.44 s from the onset of stNBM with the trough value of 79.7±1.8% (p<0.001, *vs.* prestimulus period). The stNBM-induced CBF changes were largely reduced by intraperitoneal adminstration of a mAChR antagonist atropine at a dosage ∼50 mg/kg (Figure 2B; flow peak, 105.7±2.2%, n = 5 animals, p<0.05, *vs.* positive peak of flow upon 200 µA stNBM to WT; flow trough 87.3±5.0%, p = 0.09 *vs.* negative peak of flow upon 200 µA stNBM to WT). Stimulation of a brain region 3.9 mm away from the NBM along the electrode track did not result in an increase of the CBF (Figure 2D, flow peak, 101.4±1.3%, n = 9, p = 0.30, *vs.* pre-stimulus period) while a smaller downward signal was present (flow trough, 89.8±2.4%, p<0.001, *vs.* prestimulus period). Interestingly, the initial increase of CBF was not present with a weaker stNBM (stimulation intensity: 50 µA) whereas the downward component remained (Figure 2C, n = 5 animals, 75.0±4.2%, p<0.001, *vs.* prestimulus period). Together, these experiments suggested that stNBM induces biphasic CBF changes mediated in part by cholinergic signaling.

In order to assess if astrocytic Ca^2+^ signaling plays a role in this stNBM-triggered changes of CBF, we examined the experiment using IP_3_R2-KO mice (Figure 2E, n = 13). Overall, the stNBM-triggered CBF changes were similar to C57BL/6J mice both in time course and magnitude (peak time 1.67±0.05 s; flow peak 115.3±2.4%, p<0.001, *vs.* prestimulus period; trough time 5.29±0.16 s; flow trough 76.0±2.1%, p<0.001, *vs.* prestimulus period). The positive peak values of WT and KO mice upon 200 µA stNBM were not significantly different, while they are significantly larger than that of outside-NBM or atropine experiments (p<0.05, one-way ANOVA followed by LSD test). The CBF changes after stNBM with 50 µA were similar to C57BL/6J as well (Figure 2F, flow trough 74.2±4.4%, p<0.001, *vs.* prestimulus period).

**Figure 3:**
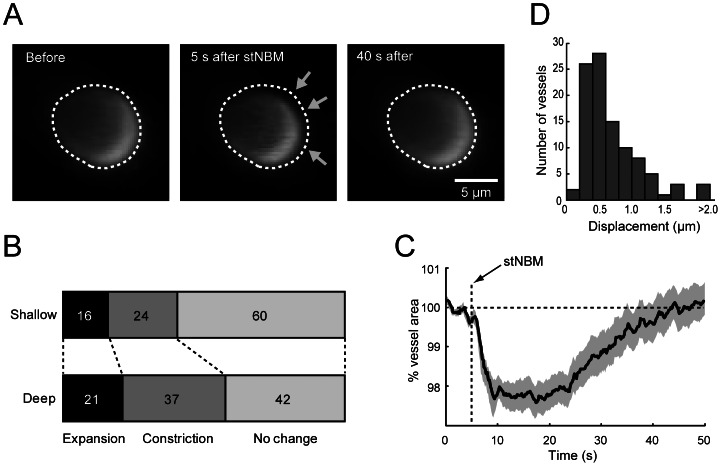
Dynamical changes of cortical arterioles by stNBM. (**A**) *In vivo* two-photon imaging of an arteriole in the somatosensory cortex upon stNBM. The arteriole is imaged at the pial depth of 200 µm. Arrows point to the region where constriction is evident. (**B**) Distribution of responses of arteriole area to stNBM. Shallower (pial depth <100 µm) arteriole were less susceptible to diameter changes than deeper arterioles (pial depth: 100∼300 µm). Numbers are in percent. (**C**) The time course of vessel area change in response to stNBM for the vessel eliciting constriction. Shaded area represents s.e.m. (**D**) Horizontal movement of imaged vessels associated with stNBM is quantified by plotting a histogram of the maximum displacement within 3 s after stNBM for each imaged arteriole.

As laser Doppler flowmetry (LDF) measures an average CBF around the laser irradiated area (i.e. a 1 mm radius hemisphere), we investigated diameter changes of cerebral arterioles by two-photon microscopy. In a proportion of the monitored arterioles, visible constrictions were observed in response to stNBM (Figure 3A). We imaged 101 arterioles in 16 C57BL/6J mice (49 arterioles at shallow pial depths of <100 µm; 52 at deep pial depths of 100∼300 µm). A large proportion of monitored arterioles (24% and 37% for “shallow” and “deep”; Figure 3B) elicited constrictions five seconds after stNBM. The time course of vessel cross-section area change for constricted arterioles is plotted in Figure 3C. The vessel cross-section area returned to the resting condition in ∼40 seconds, which roughly corresponds to the period of CBF recovery from negative overshoot upon stNBM. Arterioles that dilated in response to stNBM were heterogeneous in time course and diameter change. In some experiments, we noticed that the imaging plane transiently shifted within three seconds after stNBM. As LDF is sensitive to physical motion of measured objects, we suspected that this brain movement could contribute to the biphasic signal observed after stNBM. We quantified the maximum displacement of horizontal plane within three seconds after stNBM in Figure 3D. As a result, all imaging session had displacements smaller than three micrometer, except for one that moved five micrometer. We simulated the movement by moving the laser Doppler probe laterally (20∼50 µm) or vertically (12 µm) to the cortex in 1∼3 sec by an electronic manipulator, and confirmed that the effect does not contribute significantly to the LDF signal (data not shown). These results support the idea that the large negative LDF signal after stNBM is mediated in part by constriction of arterioles.

**Figure 4:**
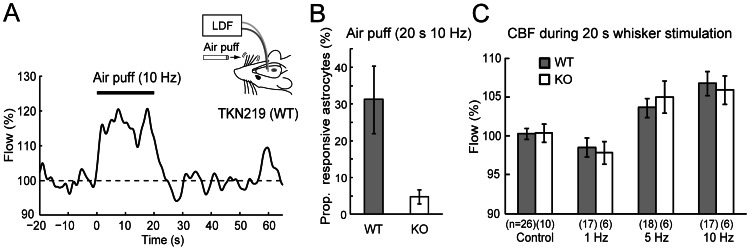
Whisker-evoked CBF changes measured by laser Doppler flowmetry from the barrel cortex. (**A**) Single example trace of 10 Hz air puff stimulation experiment. The recording was made from a WT mouse. (**B**) Proportion of barrel cortex astrocytes that elicited Ca^2+^ elevations after whisker stimulation (10 Hz, 20 s) for WT (n = 3) and IP_3_R2 KO (n = 3). (**C**) Mean CBF of WT (black) and IP_3_R2 mice (white) during 20 s whisker stimulation was compared. The number of tested animal for each stimulation paradigm is shown in parenthesis. Whisker stimulation with 5 or 10 Hz significantly increased cerebral blood flow in WT and KO when compared with prestimulus period. No significant differences in CBF response were observed between WT and KO in any of the stimulation paradigms.

Finally, we investigated functional hyperemia by stimulating whiskers contralateral to the recording site with air puffs of various frequencies. As previously documented by others, repetitive deflections of whiskers induced an increase of CBF in the primary somatosensory cortex of C57BL/6J mice (Figure 4A for an example). In a separate set of experiments, we observed that 31±9% of the imaged astrocytes elicit Ca^2+^ surges induced by the whisker stimulation whereas astrocytic Ca^2+^ surges were rare (5±2%) in IP_3_R2-KO (Figure 4B, n = 3 animals for each genotype, p<0.05). Both 5 Hz and 10 Hz stimulation paradigms induced increases of CBF during 20 seconds of stimulation (5 Hz: n = 18 animals, CBF during stimulation = 103.6±1.2%, p<0.01, *vs.* prestimulus period; 10 Hz: n = 17 animals, CBF = 106.8±1.6%, p<0.001, *vs.* prestimulus period; Figure 4C). Notably, IP_3_R2-KO mice also elicited CBF increases for both 5 Hz and 10 Hz stimulation paradigms (5 Hz: n = 6 animals, CBF = 105.0±2.1%, p<0.05, *vs.* prestimulus period; 10 Hz: n = 6 animals, CBF = 105.9±1.9%, p<0.01, *vs.* prestimulus period). The CBF during 5 Hz or 10 Hz whisker stimulation was significantly higher than control (i.e. no stimulation period) or during 1 Hz whisker stimulation. Notably, there was no significant difference between WT and KO (one-way ANOVA, followed by LSD test). The same analysis showed that 1 Hz stimulation did not result in significant changes of CBF for both genotypes. These experiments showed that whisker stimulation-triggered hyperemia is also preserved in the absence of cytosolic Ca^2+^ elevations in astrocytes.

## Discussion

Involvement of astrocytes in CBF regulation has been proposed in the past decades [17], [18]. Studies from multiple laboratories have shown that astrocytic Ca^2+^ surges and the nearby arteriole diameters are related both *in vitro* [5], [6], [8], [19] and *in vivo* [7], [20]. These CBF modulations can take form of constriction or dilation depending on the tissue oxygenation level [21] and the magnitude of astrocytic Ca^2+^ surge [22]. Indeed, some of the previous studies have confirmed causal relationships of astrocytic Ca^2+^ surges and the contacted arteriole diameter changes by Ca^2+^ uncaging in the astrocyte. More recently, a study showed that cytosolic phospholipase A_2_α and IP_3_R2 are critical components of mGluR-triggered astrocytic modulation of arteriole diameter changes [23]. Despite these findings, the current study demonstrates that the CBF changes caused by brief basal forebrain stimulation or whisker stimulation seem to persist in IP_3_R2-KO mice. As we and others have demonstrated that cytosolic Ca^2+^ surges in the somata and processes are absent in astrocytes of IP_3_R2-KO mice [11], [15], [24], our results propose that astrocytic Ca^2+^ surges or IP_3_R2 are not critical in these types of functional hyperemia.

Recently, we and others showed that paired stimulation of cholinergic nucleus and sensory stimulation leads to an enhancement of sensory response in the corresponding sensory cortex via astrocytic Ca^2+^ signaling, as such an event does not occur in IP_3_R2-KO mice [11], [25]. Accordingly, we suggested that modulation of synaptic plasticity could occur by Ca^2+^-dependent gliotransmission. As NBM stimulation induces CBF changes, it has remained possible that the CBF increase could also contribute to a favorable condition for synaptic plasticity induction by increased supply of, for instance, glucose, oxygen, or insulin-like growth factors. Conversely, the lack of the plasticity in IP_3_R2-KO could have been explained by a lack of astrocytic Ca^2+^ mediated CBF increase. Our current results that NBM-triggered CBF increases were seen in both IP_3_R2-KO and C57BL/6J suggests that such possibility is unlikely.

Assuming that astrocytic Ca^2+^ surges have little influence in the NBM- or whisker-triggered hemodynamical changes, what could be the mechanism? There are at least a few candidates. First, volume transmission of acetylcholine could directly stimulate contractile cells such as smooth muscle cells and pericytes as they express muscarinic receptors [26], [27]. Second, discharge activity of neurons and the resulting synaptic activity elevate extracellular K^+^ concentrations and it may signal nearby smooth muscle cells [28]. Third, there may be some signaling cascades independent of intracellular Ca^2+^ that could be responsible for astrocyte-driven vasomodulation. Finally, nitric oxide production due to increased neuronal activity could contribute to vasodilation [29]. It is worthwhile to note that our results do not necessarily exclude potential roles of astrocytes in CBF regulation. For instance, electrical or prolonged sensory stimulation may result in an unsual concentration of extrasynaptic neurotransmitter, activating neurovascular coupling pathways that bypass astrocytes. Another concern is potential homeostatic changes due to unconditional knockout of IP_3_R2. As floxed IP_3_R2 mice are now made and successfully used [25], these mice may be utilized to dispel adverse developmental effects by crossing with appropriate inducible Cre (e.g. Cre-ERT) lines. Alternatively, acute overexpression of phosphatases that blocks the synthesis of IP_3_ [30]–[32] or buffers of IP_3_ [33]–[36] could be used to minimize the compensatory action in future studies.

## Materials and Methods

### Ethics Statement

All animal experiment procedures were carried out in accordance with the guidelines of the Japanese Neuroscience Society. The protocol was approved by the RIKEN Institutional Animal Care and Use Committee (Protocol Number: H23-2-207).

### Laser Doppler flowmetry

Adult mice (older than nine weeks) were anesthetized by urethane (1.4 g/kg) and rigidly fixed in a stereotaxic apparatus with a heat-pad that maintained the body temperature (37°C). A bipolar stimulation electrode was inserted with an angle of 56° through a small (∼2 mm diameter, ML: 1.5 mm ipsilateral to the laser Doppler probe, AP: −3.6 mm) craniotomy and slowly progressed to the NBM (∼5.9 mm, diagonally). Successful stimulation of NBM was confirmed electrophysiologically and histochemically as described previously [11]. The fiber optic probe of a laser Doppler flowmetry (FLO-C1, Omegawave) was placed above the thinned-skull at the primary somatosensory cortex corresponding to the barrel area (Figure 1A). The “flow” output of the flowmetry device was recorded as CBF at a sampling rate of 10 kHz. Single-train electrical stimulation of the NBM (stNBM, 100 Hz, 0.5 ms pulse width, 50 pulses, 200 µA) was made using a stimulus isolator (ISO Flex driven by Master-8, AMPI). In some experiments, air-puff stimulations to whiskers were made on the contralateral side to the flowmetry at various frequencies (∼70 kPa, 5 ms pulse width, 20 s duration, 1, 5, and 10 Hz) using a pneumatic pico pump (PV830, WPI). For data analysis, CBF signals were lowpass filtered at 4 Hz to reduce the effect of pulsation.

### In vivo two-photon imaging

Two-photon imaging of vasculature was performed with urethane-anesthetized adult mice (as above) using an Olympus FV1000 laser scanning microscope (LUMPlanFl/IR 40×, wavelength 810 nm). Cerebral arteries and arterioles in the barrel area of the primary somatosensory cortex were labeled by topical application of Alexa Fluor 633 (50 µM in HEPES ringer solution, removed and washed after 10–15 minutes), as this dye was previously described to label cerebral arteries [37]. In addition, the serum was labeled by intravenous injection of FITC-dextran (2M Da) as described previously [38], [39]. For analysis of vessel diameter changes, arterioles that travel vertically yielding near-circular cross-sections were sampled.

For astrocytic Ca^2+^ imaging experiments, the Ca^2+^ indicator Fluo-4 AM was used as described previously [11]. Olympus FV1000 or Thorlabs B-Scope (XLPlan N 25×) was used to monitor Ca^2+^ levels of cortical astrocytes in response to stNBM or whisker stimulation, respectively (wavelength 820 nm).

Measured values are expressed as mean ± standard error of the mean (SEM) throughout the manuscript. The mean of CBF during 20 s preceding NBM- or whisker-stimulation was assigned as baseline. Two tailed *t*-tests were used for comparisons of two population means. For other multiple population comparisons, one-way analysis of variance (ANOVA) was performed, followed by the least significant difference (LSD) test. Data analysis was done with custom software using MATLAB (Mathworks).
